# Global Association of Cause-specific Mortality between the Major Gastrointestinal Cancers and Parkinson’s Disease for the First Two Decades of the New Millennium

**DOI:** 10.14336/AD.2021.1016

**Published:** 2022-04-01

**Authors:** Lili Lu, Meng Dai, Christina Susanne Mullins, Clemens Schafmayer, Michael Linnebacher

**Affiliations:** Clinic of General, Thoracic, Vascular and Transplantation Surgery, Molecular Oncology and Immunotherapy, Rostock University Medical Center, Rostock, Germany

**Keywords:** Parkinson’s disease;, esophagus cancer, stomach cancer, colorectal cancer, liver cancer, pancreatic cancer

## Abstract

Parkinson’s disease (PD) and gastrointestinal (GI) cancers are both “age-related diseases” sharing several environmental risk factors, but possess opposite underlying biological mechanisms. Aim of this study was to evaluate the correlations between GI cancers and PD using national cause-specific mortality data of 183 countries extracted from the Global Health Observatory database. The association between PD- and GI cancers- (i.e. esophagus cancer, EC; stomach cancer, SC; colorectal cancer, CRC; liver cancer, LC and pancreatic cancer, PC) specific mortality on the country level was evaluated using Spearman correlation and logistic regression analysis. A global increase in mortality from 2000 to 2019 was observed in PD, CRC and PC, whereas in EC, SC and LC it decreased. We see the consistent diminishment of correlation intensities between PD and GI cancer mortalities from 2000 to 2019 as a positive development. In 2019, PD inversely correlated with CRC (*r_s_* = -0.39) and PC (*r_s_* = -0.40, all *P* < 0.001) but not with EC and SC. Of note, an exceptionally positive correlation of PD with LC (*r_s_* = 0.26, *P* < 0.001) and its two hepatitis B and C virus-associated subtypes was revealed. Logistic regression analysis further determined that PD associated negatively with CRC (OR = 0.25) and PC (OR = 0.21, both *P* < 0.001), but positively with LC (OR = 2.27, *P* = 0.007). Consequently, future research aiming to unravel the functional biological link between neurodegeneration, hepatitis and tumor development holds great potential for developing novel therapeutics.

Parkinson’s disease (PD) is a major progressive neurodegenerative disease, and its global age-standardized prevalence increased by 21.7% from 1990 to 2016 [1]. Cancer behaves biologically opposite and is characterized by uncontrolled cellular multiplication, having caused 4.8 million new cases and 3.4 million deaths worldwide in 2018 [2]. In addition to mortality, PD and cancer substantially cause morbidity and are both prototypic diseases strongly affecting aging populations. Shared environmental risk factors, besides aging [3], are obesity, diabetes, alcohol abuse, smoking and possibly the gut microbiome [4-6]. Epidemiological data suggest an inverse correlation between PD and cancer [7], even a significantly lower cancer occurrence in PD patients [8, 9]. In addition to the opposing biological mechanisms of both diseases, pharmacological treatment seems to contribute to this interrelationship, too [9].

In the present study, we wanted to clarify this by evaluating the correlations between mortality from five major gastrointestinal (GI) cancers (esophagus cancer, EC; stomach cancer, SC; colorectal cancer, CRC; liver cancer, LC and pancreatic cancer, PC) and from PD using national cause-specific mortality data; these were extracted from the Global Health Observatory database which allowed us to include 183 countries [10].

**Figure 1.**
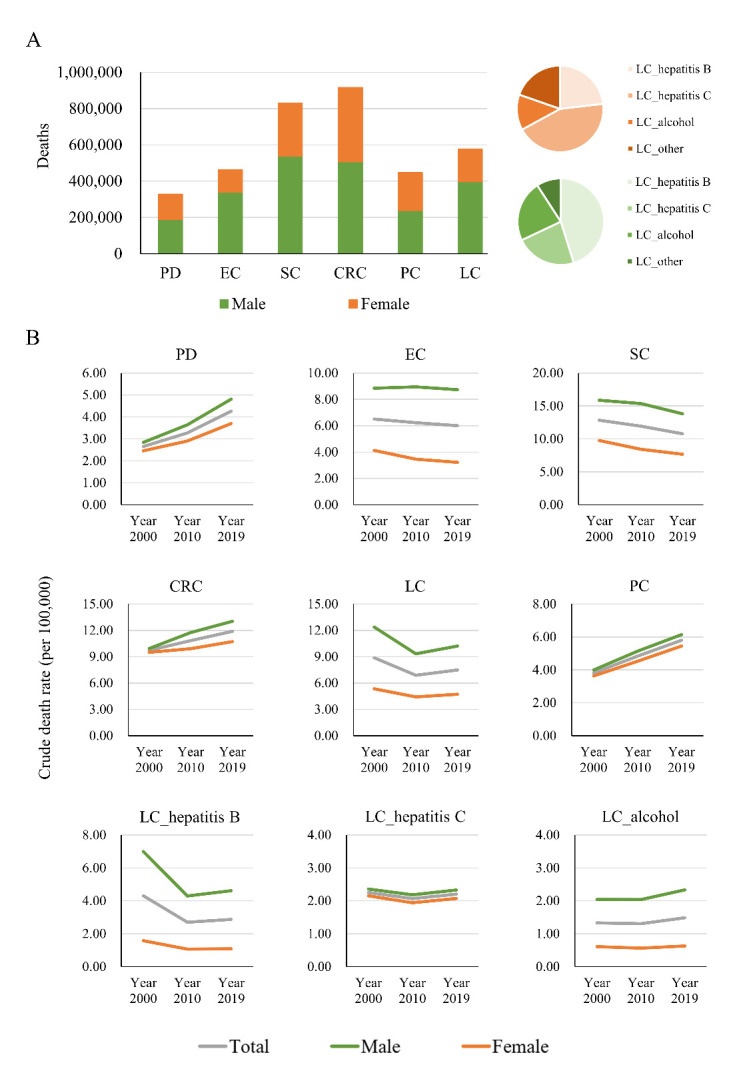
Death toll from PD and GI cancers in the last two decades. Death number of PD and GI cancers for both genders in 2019 (A); crude death rate of PD and GI cancers for both genders in the years 2000, 2010 and 2019 (B).

## METHODS

The global crude death rate was calculated as follows: death number / total population and is presented as rate per 100,000 inhabitants. The national mortality is given as age-standard mortality per 100,000 inhabitants [10-12]. We included the main GI cancers: EC, SC, CRC, LC and PC. Moreover, the three subgroups of LC (LC secondary to hepatitis B (LC_hB), to hepatitis C (LC_hC) and to alcohol abuse (LC_alcohol)) were investigated separately. The analysis variables included year of estimate (2000, 2010 and 2019) and gender (male and female).

**Figure 2.**
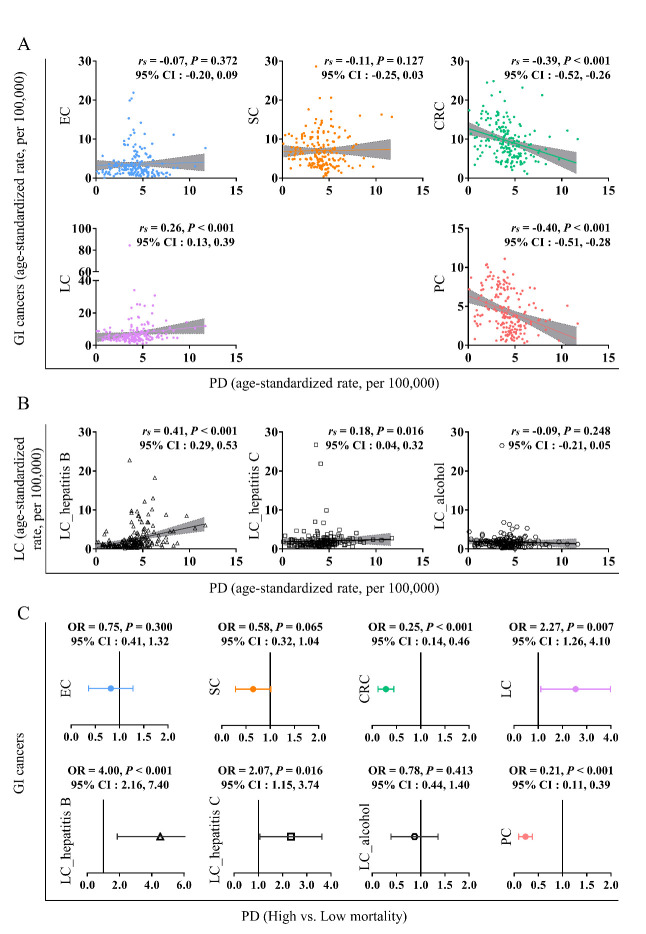
Association between PD and GI cancers in the year 2019. Correlation between PD and GI cancers (A); correlation between PD and three subtypes of LC (B); effects of PD as a risk factor for GI cancers (C).

The correlation between PD- and GI cancers- specific mortality was evaluated using Spearman correlation analysis and is presented by correlation coefficient, *r_s_* and 95% confidence intervals (CIs). In order to define the risk factors, all countries were divided into two groups (high- versus low-mortality countries) based on the median value of PD- and GI cancers- specific mortality (cutoff value of EC: 2.45; SC: 6.00; CRC: 8.70; LC: 5.40; LC_hB: 1.40; LC_hC: 1.50; LC_alcohol: 1.40; PC: 3.70 and PD: 4.10 per 100,000); then evaluated the impact of PD on the higher mortality of GI cancers at a national level using logistic regression analysis and shown as odds ratios (ORs) and 95% CIs. Statistical analyses were performed by IBM SPSS Statistics, version 20.0, and *P* < 0.05 was considered statistically significant.

## RESULTS

For the year 2019, 328,645 deaths from PD, 462,995 deaths for EC, 830,682 for SC, 916,166 for CRC, 577,430 for LC and 447,208 for PC were reported globally, with males always being more frequently affected than females (Fig. 1A). For LC, 38.29% and 19.87% deaths are secondary to hepatitis B and alcohol, respectively. Whereas 29.39% LC deaths are secondary to hepatitis C and solely in this pathology, females are more often affected (Fig. 1A).

The global crude death rates of PD for the years 2000, 2010 and 2019 showed an increasing trend for both men and women. Increasing numbers were also seen in CRC and PC (Fig. 1B), but mortality of EC and SC decreased. For LC, a strong reduction was documented from 2000 to 2010, followed by a slight increase until the year 2019 (Fig. 1B).

Correlating the national cause-specific mortalities of PD and GI cancers from 183 countries for the year 2019, no significant factor was found for EC and SC with PD, but PD inversely correlated with CRC (*r_s_* = -0.39, 95% CI: -0.52, -0.26; *P* < 0.001) and PC (*r_s_* = -0.40, 95% CI: -0.51, -0.28; *P* < 0.001) (Fig. 2A).

Contrarily, and a little unexpected, an exceptionally strong positive correlation with PD was revealed for LC (*r_s_* = 0.26, 95% CI: 0.13, 0.39; *P* < 0.001) (Fig. 2A). Since LC development is mainly triggered by three differing causes, we next correlated mortality between PD and the LC pathogenetic subtypes. Positive correlations with the two virus-associated subtypes LC_hB (*r_s_* = 0.41, 95% CI: 0.29, 0.53; *P* < 0.001) and LC_hC (*r_s_* = 0.18, 95% CI: 0.04, 0.32; *P* = 0.016) were observed, but not with LC_alcohol (*r_s_* = -0.09, 95% CI: -0.21, 0.05; *P* = 0.248) (Fig. 2B).

**Figure 3.**
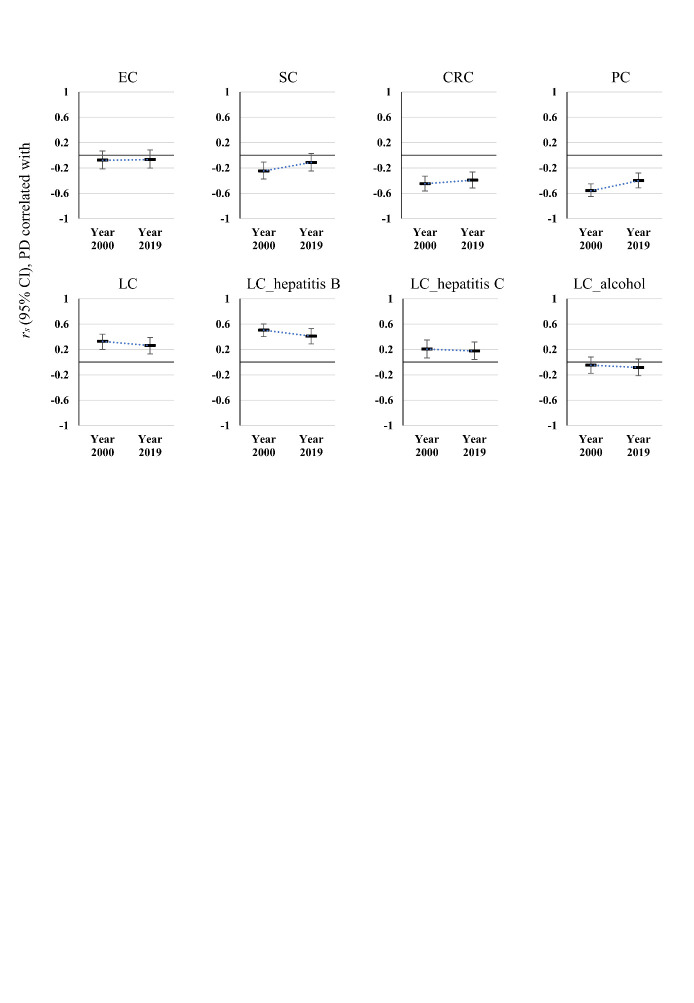
Comparison of correlation coefficients (*r_s_*) of PD and GI cancers between 2000 and 2019.

In addition, the logistic regression analyses further determined that countries with high PD mortality had a decreased mortality risk of CRC (OR = 0.25, 95% CI: 0.14, 0.46; *P* < 0.001) and PC (OR = 0.21, 95% CI: 0.11, 0.39; *P* < 0.001), but an increased risk of LC (OR = 2.27, 95% CI: 1.26, 4.10; *P* = 0.007) (Fig. 2C). In more detail, this positive correlation with high PD mortality was also seen in countries with high mortality of the two virus-associated subtypes LC_hB (OR = 4.00, 95% CI: 2.16, 7.40; *P* < 0.001) and LC_hC (OR = 2.07, 95% CI: 1.15, 3.74; *P* = 0.016) (Fig. 2C).

Notably, the intensities of the correlations between PD and GI cancer mortalities consistently diminished from 2000 to 2019. Precisely, the correlation coefficient of PD and PC rose from -0.56 (95% CI: -0.65, -0.45; *P* < 0.001) in 2000 to -0.40 (95% CI: -0.51, -0.28; *P* < 0.001) in 2019, the coefficient of PD and CRC from -0.45 (95% CI: -0.56, -0.33; *P* < 0.001) to -0.39 (95% CI: -0.52, -0.26; *P* < 0.001); whereas the correlation coefficient of PD and LC diminished from 0.33 (95% CI: 0.20, 0.44; *P* < 0.001) to 0.26 (95% CI: 0.13, 0.39; *P* < 0.001) within these two decades (Fig. 3). This decrease in correlation intensity pertained to two subgroups of LC secondary to hepatitis (LC_hB: *r_s_* = 0.51, 95% CI: 0.40, 0.60 in 2000 and *r_s_* = 0.41, 95% CI: 0.29, 0.53 in 2019, all *P* < 0.001; LC_hC: *r_s_* = 0.21, 95% CI: 0.07, 0.35; *P* = 0.005 in 2000; *r_s_* = 0.18, 95% CI: 0.04, 0.32; *P* = 0.016 in 2019) (Fig. 3). For SC, the significant inverse correlation with PD observed in the year 2000 (*r_s_* = -0.25, 95% CI: -0.37, -0.11; *P* = 0.001) was even lost in the year 2019 (*r_s_* = -0.11, 95% CI: -0.25, 0.03; *P* = 0.127) (Fig. 3).

## DISCUSSION

In the present study, we observed that PD-specific mortality was inversely correlated with the mortality of CRC and PC; even countries with high PD mortality were associated with the lower mortality of CRC and PC. These findings are for the most part consistent with previous reports including a nationwide population-based cohort study describing that PD patients had a decreased risk of SC, CRC, LC as well as PC [13]. However, and a little unexpected, a positive association between PD and LC was revealed in this study. In detail, this positive association with PD was mostly seen in the two virus-associated pathogenetic subtypes: LC_hB and LC_hC.

An Israeli study from 2019 also described that hepatitis B viruses (HBV) and hepatitis C viruses (HCV) were risk factors for PD development [14], while a large Taiwanese cohort study concluded that HCV-positive, but not HBV-infected patients have an enhanced risk of developing PD [15]. Mechanistically, HCV has been shown to cause the loss of dopaminergic neurons by inducing neuro-inflammation [16]. Taking into account the even stronger correlation, we observed between HBV and PD, it seems reasonable to suggest that HBV is also a likely co-risk factor for PD development. In addition to neuro-inflammation, neuronal cell damage and cell death could be induced directly by viral replication or indirectly by activating further innate immune response mechanisms [17].

LC_alcohol tended to be weakly associated inversely with PD. This can be interpreted as supporting an old hypothesis stating that moderate alcohol consumption might be protective for PD; which might be attributable, for example, to the urate-elevating effects of beer [18].

In addition, likely explanations for the decrease in correlation intensity are improvements in therapeutical management of PD, especially in the developed countries, combined with a strong increase of GI cancer numbers in developing countries [1, 19].

We found no significant correlation between EC and PD. This might be due to the incapability to subdivide EC into the two main subtypes of esophageal squamous cell carcinoma and adenocarcinoma, since the Global Health Observatory database does not provide this classification.

Despite this clear limitation of the present study, this is the first report correlating PD and GI cancers mortalities on the global scale. In sum, our data are in favor of the hypothesis of opposing biological processes driving the two types of diseases. As we mentioned before, the neurodegenerative disease gradually leads to the death of neurons, while cancer is characterized by ceaseless cell proliferation [9].

Given the biologically opposing disease mechanisms, future research must address the “chicken or egg” causality dilemma of PD’s correlation with most GI cancers. Albeit successful HBV vaccination plus the dramatically improved clinical management of HCV-infected patients reduced the number of novel virus-associated LC cases, the positive correlation of the latter with PD warrants detailed analysis. Basing on these data, it is only logical to conclude, that viral hepatitis prevention thus might, on the longer run, help reduce the number of people affected by PD.

Finally, both lines of future research aiming at unraveling the functional biological link between neurodegeneration, viral infection and tumor development hold great potential for developing novel therapeutic concepts.
